# Three-Year Echocardiographic Follow-Up in Outpatients with Systemic Arterial Hypertension: An Observational Cohort Study

**DOI:** 10.3390/jcm14165812

**Published:** 2025-08-17

**Authors:** Tiberiu-Liviu Dragomir, Minodora Andor, Petrinela Daliu, Norberth-Istvan Varga, Razvan Susan, Razvan Mihai Horhat, Laura Nicolescu

**Affiliations:** 1 Medical Semiology II Discipline, Internal Medicine Department, “Victor Babes” University of Medicine and Pharmacy, Eftimie Murgu Square No. 2, 300041 Timisoara, Romania; dragomir.tiberiu@umft.ro (T.-L.D.); andor.minodora@umft.ro (M.A.); 2 Doctoral School, “Victor Babes” University of Medicine and Pharmacy, Eftimie Murgu Square No. 2, 300041 Timisoara, Romania; petrinela.daliu@umft.ro; 3 Department of Family Medicine, Centre for Preventive Medicine, “Victor Babes” University of Medicine and Pharmacy, Eftimie Murgu Square No. 2, 300041 Timisoara, Romania; 4 Department of Restorative Dentistry, Faculty of Dentistry, “Victor Babes” University of Medicine and Pharmacy, Eftimie Murgu Square No. 2, 300041 Timisoara, Romania; horhat.razvan@umft.ro; 5 Doctoral School, Faculty of Medicine, “Vasile Goldis” Western University, Bulevardul Revolutiei 94, 310025 Arad, Romania; laura_dsp@yahoo.com

**Keywords:** hypertension, echocardiography, left ventricular hypertrophy, left ventricular mass, left ventricular ejection fraction, interventricular sept, left ventricle posterior wall, diastolic disfunction

## Abstract

**Background/Objectives**: Systemic arterial hypertension is a prevalent condition associated with adverse cardiac remodeling. Echocardiography plays a crucial role in assessing cardiac structure and function in hypertensive patients. This study aimed to evaluate the changes in echocardiographic parameters in hypertensive patients over a 3-year follow-up period and assess the impact of blood pressure control and antihypertensive medication use on these changes. **Methods**: This observational cohort study included 131 adult patients with systemic arterial hypertension who underwent annual echocardiographic assessments. Statistical analyses included paired and unpaired comparisons, regression modeling, and subgroup analyses by BP control and lifestyle behavior. **Results**: Over the 3-year follow-up, mean left ventricular mass (LVM) increased significantly from 173.99 ± 59.33 g to 183.26 ± 64.19 g (*p* = 0.018), and the prevalence of LV hypertrophy rose from 29.0% to 40.5% (*p* = 0.021). Patients with uncontrolled blood pressure at the final visit had significantly greater interventricular septum and posterior wall thicknesses (*p* = 0.009 and *p* = 0.012, respectively), but no statistically significant difference in ΔLVM. Those who adopted more healthy lifestyle behaviors showed a dose-dependent reduction in LVM progression, with each additional lifestyle improvement associated with a −3.25 g change in ΔLVM (*p* = 0.01). Multivariable linear regression identified baseline LVM, sex, and lifestyle score as independent predictors of 3-year LVM change (model R^2^ = 0.318). **Conclusions**: Our findings indicate that long-term cardiac remodeling may continue in hypertensive patients despite treatment, particularly in the presence of suboptimal BP control. Sustained lifestyle improvements were independently associated with attenuation of LVM progression. These results underscore the importance of integrating behavioral interventions alongside pharmacologic therapy in routine hypertension management.

## 1. Introduction

Systemic arterial hypertension, defined as a sustained elevation of systemic arterial blood pressure, is a prevalent chronic disease and a major risk factor for cardiovascular morbidity and mortality [1,2,3]. Current guidelines define arterial hypertension as a systolic blood pressure (SBP) ≥ 140 mmHg and/or a diastolic blood pressure (DBP) ≥ 90 mmHg [1,2,3,4,5]. The prevalence of arterial hypertension is alarmingly high, affecting an estimated 1.28 billion adults worldwide, with a significant proportion remaining undiagnosed and inadequately controlled [6]. This chronic condition imposes a sustained hemodynamic load on the heart, leading to adverse cardiac remodeling, including left ventricular hypertrophy (LVH), collectively termed hypertensive heart disease (HHD) [7,8]. Echocardiography plays a crucial role in the evaluation of HHD, providing a non-invasive means of assessing cardiac structure and function [9,10].

Chronic systemic arterial hypertension induces cardiac remodeling through increased afterload, which triggers compensatory myocardial hypertrophy and fibrosis [7,8,9,10,11,12]. This results in increased LVM, primarily due to thickening of the interventricular septum (IVS) and left ventricular posterior wall (LVPW), contributing to LVH [13,14]. Left ventricular internal dimension at end-diastole (LVIDd) may also increase as the ventricle adapts to sustained pressure. Initially, left ventricular ejection fraction (LVEF) remains preserved, but prolonged remodeling can impair systolic function [15,16]. Diastolic dysfunction, reflected by a reduced E/A ratio, often develops due to myocardial stiffening, further compromising cardiac performance [17]. These changes, if unchecked, elevate the risk of heart failure and cardiovascular events, underscoring the importance of monitoring echocardiographic parameters in hypertensive patients.

Despite extensive research using echocardiography in hypertensive heart disease, several nuances of cardiac remodeling in hypertension remain under active investigation. The benefits of antihypertensive treatment on reversing LV hypertrophy and improving diastolic function are well established. A recent meta-analysis demonstrated that all major antihypertensive drug classes and their combinations can lead to significant reductions in LV mass (on the order of ~5% decrease on average) [18]. Furthermore, comparative real-world studies suggest that achieving blood pressure control is key: for instance, adding either a thiazide diuretic or a calcium-channel blocker to a renin–angiotensin system blocker yielded similar degrees of LV mass regression over 6 months [19]. Nevertheless, the optimal approach to maximize long-term remodeling benefits is still being clarified.

Another important consideration is the prognostic significance of these echocardiographic changes. High LV mass is a known risk factor for adverse outcomes in hypertension, yet whether treatment-induced changes in LVM, LVEF, or other echo parameters translate into improved prognosis is not fully settled [20]. Some cohort studies have demonstrated that persistent or new-onset LVH during therapy portends higher cardiovascular risk, whereas regression of LVH is associated with fewer cardiac events [20,21,22]. Other research suggests that LVH regression may primarily reflect effective blood pressure control and might not independently improve outcomes [23]. Given these nuanced and sometimes conflicting findings, ongoing real-world research plays a vital role in refining our understanding of hypertensive cardiac remodeling.

The primary aim of our study was to assess the longitudinal changes in echocardiographic parameters over a 3-year follow-up in a cohort of hypertensive patients receiving routine outpatient care. As a secondary objective, we examined whether the degree of blood pressure control, as assessed by annual 24 h ambulatory monitoring, influenced the magnitude of these echocardiographic changes. Additionally, we investigated the potential associations between different classes and combinations of antihypertensive medications and cardiac remodeling parameters. Finally, we explored the impact of sustained lifestyle modifications, including smoking cessation, regular physical activity, and weight-reducing dietary changes, on left ventricular structure over time. This real-world perspective aims to clarify the interplay between pharmacologic and behavioral factors in shaping cardiac remodeling, and to complement the existing literature on long-term cardiovascular outcomes in systemic arterial hypertension.

## 2. Materials and Methods

### 2.1. Study Design and Population

This observational prospective cohort study included adult patients (age > 18 years) who had a prior diagnosis of systemic arterial hypertension, and who regularly attended the “Lifetreat” outpatient clinic in Timisoara, Romania. Adult patients were considered eligible for study enrollment if they had a diagnosis of hypertension, with or without additional cardiovascular risk factors or comorbidities, regardless of age, provided they gave written informed consent. Their data were collected prospectively over the course of three years, during routine annual check-ups, starting from the 1st of January 2022 to the 31st of December 2024. Clinical and echocardiographic data obtained at the initial visit (in other words, data collected in 2022) were defined as baseline measurements. Patients were excluded from the statistical analysis if they declined participation (no justification was required) or if they lacked complete annual data across all three consecutive years.

Prior to patient enrollment, a power analysis was conducted in order to approximate the number of patients that should be included (see the results in Section 2.3).

This study adhered to the principles of the Declaration of Helsinki. All participants provided written informed consent before enrollment in the study. The study protocol was approved by the Institutional Board of the medical institution where data were collected (Reference number 19/20.12.2021).

### 2.2. Data Collection

Data were collected prospectively, at the clinic, upon regular, annual assessments.

Demographic information included age, sex, and place of residence (rural/urban). At each recorded visit, blood pressure, weight, and height were measured, and the body mass index (BMI) was calculated. Blood pressure was assessed annually using 24 h ambulatory blood pressure monitoring (ABPM), performed at each scheduled check-up using a clinically validated oscillometric device (Mobil-O-Graph NG, IEM GmbH, Stolberg, Germany). Patients were classified as having controlled blood pressure if their 24 h average systolic BP was <140 mmHg and diastolic BP was <90 mmHg.

Furthermore, at each annual visit, the following data were collected/reassessed: smoking status, alcohol consumption, dietary habits and/or modifications, data on comorbidities, physical exercise, and medication used. Lipid profiles (total cholesterol, HDL-cholesterol) and diabetes mellitus (DM) status (defined by prior diagnosis or HbA1c > 6.5%) were extracted from fasting blood samples analyzed at the clinic’s certified laboratory using standard enzymatic assays (Roche Cobas 6000 analyzer). Antihypertensive therapy was prescribed according to the ESC/ESH recommendations at the discretion of the attending cardiologist. No study-mandated drug selection was enforced whatsoever.

Echocardiographic parameters were assessed annually by two experienced cardiologists (T-L.D. or M.A.). All examinations were performed on a Philips EPIQ 7 system (Philips Healthcare, Andover, MA, USA) equipped with an X5-1 phased-array transducer. Images were acquired in the left-lateral decubitus position and stored digitally according to ASE/EACVI 2015 recommendations. Two-dimensional (2D)-guided M-mode measurements of the interventricular septum (IVSd), left-ventricular internal diameter (LVIDd), and posterior-wall thickness (LVPWd) were obtained in parasternal long-axis at end-diastole. Left-ventricular mass (LVM) was calculated by the Devereux-corrected cube formula: LVM (g) = 0.8 × 1.04 × [(IVSd + LVIDd + LVPWd)^3^ − LVIDd^3^] + 0.6.

Left-sided valvular heart disease (VHD) data were collected from the clinical echo reports as absent/trace, mild, moderate, or severe; for analyses, we used a binary indicator of any ≥moderate left-sided VHD at baseline.

Diastolic function was assessed using the E/A ratio. Left ventricular hypertrophy (LVH) was defined as a binary variable based on IVS and LVPW measurements, according to the American Society of Echocardiography criteria [24]. End-diastolic and end-systolic volumes were traced in the apical four- and two-chamber views and left-ventricular ejection fraction (LVEF) was derived by Simpson’s biplane method of disks.

Body-surface area (BSA) was calculated with the Mosteller formula: BSA (m^2^) = √(height (cm) × weight (kg)/3600). LVM was then indexed to BSA (LVMI). Left-ventricular hypertrophy (LVH) was defined as LVMI >95 g/m^2^ in women and >115 g/m^2^ in men. Relative-wall thickness (RWT) was computed as (IVSd + LVPWd)/LVIDd, with a threshold of 0.42 to distinguish concentric versus eccentric geometry.

### 2.3. Statistical Analysis

To determine the sample size for evaluating longitudinal changes in left ventricular mass (LVM) over a 3-year period, an a priori power analysis was conducted based on an expected mean LVM change of approximately 10 g with a standard deviation of 30 g. A sample size of 126 patients was calculated to achieve 80% power at a 0.05 significance level using a paired *t*-test to approximate the Wilcoxon signed-rank test. Accounting for a 15% loss to follow-up, the target sample size was increased to 149 patients.

All statistical analyses were performed using SPSS version 26 (SPSS Inc., Chicago, IL, USA). Continuous variables were assessed for normality using the Kolmogorov–Smirnov and Shapiro–Wilk tests. Normally distributed continuous variables were compared between groups using the independent samples *t*-test, while non-normally distributed variables were compared using the Mann–Whitney U test. Changes in continuous variables over time were assessed using the paired *t*-test for normally distributed data and the Wilcoxon signed-rank test for non-normally distributed data. Change scores (Δ-variables) were computed as final measurement—baseline measurement (e.g., ΔLVEF = LVEF_Final − LVEF_Baseline; and ΔLVM = LVM_Final − LVM_Baseline).

For analysis, antihypertensive medications were grouped into the major classes: beta-blockers, diuretics, ACE inhibitors, ARBs, calcium channel blockers, etc. When ≥3 medication or lifestyle groups were compared, one-way ANOVA was applied if assumptions held; otherwise, the Kruskal–Wallis test was used, with Dunn–Bonferroni post hoc contrasts when appropriate.

To identify independent predictors of ΔLVM, a multiple linear regression was fitted (enter method) with baseline LVM, age, sex, BP-control status (controlled vs. uncontrolled), and lifestyle-score (0–3) as covariates. Model assumptions (linearity, homoscedasticity, normality of residuals, and multicollinearity via VIF) were verified. As an additional analysis, we re-estimated the ΔLVM model, including the valvular heart disease (VHD) indicator, with covariates selected a priori based on medical plausibility (baseline LVM, age, sex, coronary artery disease, hypertriglyceridemia, and hyperuricemia).

Results were reported as mean ± SD, median [25th–75th interquartile range], β (95% CI), or n (%), as appropriate.

For all analyses, statistical significance was defined as a two-tailed *p*-value < 0.05.

## 3. Results

### 3.1. Baseline Characteristics of Study Population

According to the power analysis results, 149 consecutive patients, who agreed to participate in this study and signed informed consent, were enrolled at the initial visit in 2022. A total of 18 patients were excluded from statistical analysis due to a lack of follow-up data, as per methodology, leaving a total population of 131 patients with systemic arterial hypertension who underwent annual echocardiographic assessments over a 3-year period.

The mean age at the first visit was 59.93 years (±15.51), with a slightly higher proportion of males (51.9%). The majority of participants resided in urban areas (63.4%). The mean BMI was 32.15 kg/m^2^ (±4.9) for males and 30.96 kg/m^2^ (±3.27) for females. Notably, none of the participants were underweight; 20.6% had a normal weight (n = 27), 24.4% were overweight (n = 32), 29.0% had grade I obesity (n = 38), 18.3% had grade II obesity (n = 24), and 7.7% had grade III obesity (n = 10).

A significant proportion of patients presented with comorbidities that are commonly associated with hypertension. Specifically, 30.5% had diabetes mellitus (n = 40), 48.1% had hypercholesterolemia (n = 63), and 37.4% had hypertriglyceridemia (n = 49). A quarter of the patients (25.2%) had a history of coronary artery disease. Angina was present in 33 patients, with the majority experiencing grade I (n = 18, 13.7%) or grade II (n = 9, 6.9%) angina, while grade III angina was observed in 5 patients (3.8%), and grade IV angina in 2 patients (1.5%). Atrial fibrillation was observed in 11.5% of the participants at baseline (n = 15). Left-sided valvular heart disease (VHD) was ≥moderate in 21 patients (mitral stenosis n = 6, mitral regurgitation n = 7, aortic stenosis n = 4, and aortic regurgitation n = 4) and mild in 28 (MS n = 8, MR n = 8, AS n = 7, and AR n = 5); the remainder had no left-sided VHD.

At the first visit, most patients were using multiple antihypertensive medications. Beta-blockers were the most commonly used class (64.9%), followed by diuretics (61.1%), angiotensin-converting enzyme (ACE) inhibitors (51.1%), angiotensin II receptor blockers (ARBs–42.7%), and calcium channel blockers (CCBs–36.6%). Over half of the participants (54.2%) were taking statins. Other medications, including antiplatelet agents (34.4%), anticoagulants (16.0%), and antiarrhythmics (9.2%), were also prescribed, reflecting the complex cardiovascular profiles of this patient population. The majority of patients (75.6%) were on combination antihypertensive therapy, with the most common combinations being beta-blocker + ACE inhibitor (n = 37), beta-blocker + diuretic (n = 33), and CCB + diuretic (n = 30).

Of the 131 patients, roughly 42% (n = 55) were categorized as having uncontrolled hypertension, on the basis of 24 h ABMP at the last (third) measurement, while 58% (n = 76) had an average BP < 140/90 mmHg per 24 h and were, therefore, included in the controlled hypertension group.

Table 1 offers an overview of the study population characteristics at baseline.

### 3.2. Baseline vs. Final Visit—Changes in Echocardiographic Parameters

All continuous echocardiographic variables were screened with the Shapiro–Wilk test; none met the assumption of normality (all *p* < 0.05). Paired comparisons were, therefore, performed with Wilcoxon signed-ranks tests throughout. For descriptive purposes, values are still presented as mean ± SD because (1) the cohort size (n = 131) is large enough for means to remain stable and clinically familiar, and (2) this format facilitates comparison with previous left-ventricular mass (LVM) regression studies that also report means. Table 2 summarizes the mean changes in echocardiographical parameters.

Mean baseline LVM was 173.99 ± 59.33 g. This corresponded to an LVMI of 92 ± 24 g/m^2^ in women and 103 ± 26 g/m^2^ in men. At year 3, mean LVM increased to 183.26 ± 64.19 g (Wilcoxon z = −2.364, *p* = 0.018). In parallel, LVMI rose to 97 ± 26 g/m^2^ in women (z = −2.21, *p* = 0.027) and 109 ± 28 g/m^2^ in men (z = −2.09, *p* = 0.037). Concentric remodeling, concentric LVH, and eccentric LVH were present in 18%, 23% and 19% of patients at baseline, shifting to 15%, 29%, and 20% at year 3 (*p* = 0.048). Baseline LVEF averaged 54.51 ± 6.57% and 54.56 ± 7.18% at follow-up; the change was not significant (z = −0.145, *p* = 0.585).

The E/A ratio declined slightly from 1.06 ± 0.41 to 1.02 ± 0.44 (z = −0.851, *p* = 0.195). Correspondingly, the prevalence of diastolic-dysfunction grades shifted from 52 (grade I) and 22 (grade II) patients at baseline to 61 and 28, respectively, at year 3. Fifteen patients with atrial fibrillation were excluded from this analysis because their transmitral flow could not be reliably assessed. LVIDd showed a statistically significant increase from 4.65 ± 0.56 cm to 4.72 ± 0.62 cm (z = −2.502, *p* = 0.012).

Interventricular-septum (IVS) thickness rose marginally from 1.026 ± 0.19 cm to 1.062 ± 0.18 cm, while posterior-wall (LVPW) thickness changed from 1.042 ± 0.16 cm to 1.050 ± 0.17 cm; neither difference reached statistical significance (IVS z = −1.603, *p* = 0.109; LVPW z = −0.572, *p* = 0.567). At baseline, 38 patients (29%) had left ventricular hypertrophy (LVH). At the third measurement, the prevalence of LVH increased to 53 patients (40.5%). McNemar’s test showed that this increase in the prevalence of LVH was statistically significant (χ^2^(1) = 5.333, *p* = 0.021).

### 3.3. Association Between Blood Pressure Control and Changes in Echocardiographic Parameters

Based on 24 h ambulatory blood pressure monitoring (ABPM) at the final (third-year) assessment, patients were categorized into two groups: controlled blood pressure (n = 76, 58%) and uncontrolled blood pressure (n = 55, 42%). To assess the association between blood pressure control and changes in echocardiographic parameters, we first examined the distributions of the change scores for each parameter. Based on the results of the normality of distribution tests, we used independent samples *t*-tests to compare changes in LVM and LVEF between the controlled and uncontrolled blood pressure groups, and Mann–Whitney U tests for changes in E/A ratio, IVS, and LVPW.

The *t*-tests revealed no statistically significant difference in ΔLVM (t(85) = 0.740, *p* = 0.071) or ΔLVEF (t(85) = 1.521, *p* = 0.132) between the two groups. The mean change in LVM was 13.47 g in the uncontrolled group and 7.49 g in the controlled group. The mean change in LVEF was 1.54% in the uncontrolled group and −0.59% in the controlled group. Although the difference in LVM did not reach statistical significance, the result suggested a trend toward higher increases in LVM among patients with uncontrolled blood pressure.

The Mann–Whitney U tests showed no statistically significant difference in the change in E/A ratio between the two groups (U = 696.500, *p* = 0.371). However, there was a statistically significant difference in both IVS (U = 514.000, *p* = 0.009) and LVPW (U = 551.000, *p* = 0.019), indicating that patients with uncontrolled blood pressure experienced a greater increase in both interventricular septum and posterior wall thickness over time compared to those with controlled blood pressure.

### 3.4. Association Between Classes of Antihypertensive Medication and Changes in Echocardiographic Parameters

Analysis of antihypertensive medication use revealed that monotherapy was rare in our study population. A total of 18 patients were taking ACE inhibitors, and 14 used diuretics as monotherapy. The majority of patients (75.6%) were on combination antihypertensive therapy, with the most common combinations being beta-blocker + ACE inhibitor (n = 37), beta-blocker + diuretic (n = 33), and CCB + diuretic (n = 30). Less frequent combinations included triple therapy with beta-blockers, ACE inhibitors, and diuretics (n = 7), triple therapy with calcium channel blockers, ACE inhibitors, and diuretics (n = 8), and quadruple therapy with all four classes (n = 5).

We initially considered a one-way ANOVA to compare the change in left ventricular mass between different medication groups. However, Levene’s test indicated a violation of the assumption of equal variances (*p* = 0.044). Therefore, a Kruskal–Wallis test was conducted, and the results showed no statistically significant difference in ΔLVM between the groups (χ2(4) = 2.285, *p* = 0.683).

A one-way ANOVA was conducted to compare the change in LVEF between different medication groups. The assumption of homogeneity of variances was not violated (Levene’s test, *p* = 0.558). The results showed no statistically significant difference in ΔLVEF between the medication groups (F(4, 82) = 0.311, *p* = 0.870).

### 3.5. Impact of Lifestyle Changes on Echocardiographic Remodeling

To explore the influence of lifestyle modifications on cardiac remodeling, patients were scored from zero to three based on the number of sustained healthy behavior changes reported over the 3-year study period. One point was assigned for each of the following: (1) smoking cessation, (2) regular exercise (an average of 30 min of easy physical exercise per day, or approx. 3 h/week), and (3) dietary changes resulting in weight loss equivalent to at least one grade of obesity.

Out of 66 initial smokers, 24 gave up smoking by the time of the third check-up. Out of 72 obese patients (all three grades of obesity), 29 patients successfully implemented dietary changes that resulted in weight loss, as described above. A total of 38 patients out of 131 reported consistency in physical exercise. A total of 19 patients implemented all three changes (score = 3), 27 patients adopted two changes (score = 2), and 24 adopted one change (score = 1).

A Kruskal–Wallis test was conducted to compare the change in left ventricular mass (ΔLVM) across the four lifestyle score groups. The test revealed a statistically significant difference between the groups (χ^2^(3) = 8.91, *p* = 0.031). Patients with higher lifestyle scores demonstrated greater reductions in LVM over the 3-year period. The median ΔLVM was +7.5 g [+2.1, +9.9] in the no-change group (score = 0), +1.0 g [−2.3, +3.3] in the score 1 group, −2.2 g [−6.8, +0.9] in the score 2 group, and −4.3 g [−8.7, +0.2] in the score 3 group. These findings suggest a potential dose–response relationship between the number of sustained healthy behaviors and the degree of favorable cardiac remodeling. Table 3 summarizes these findings.

To translate these rank-based differences into a continuous estimate, we fitted a multivariable linear model: each one-point improvement in the lifestyle score corresponded to a 3.3 g reduction in ΔLVM (β = −3.3 g; 95% CI −6.1 to −0.5; *p* = 0.02), mirroring the ~11 g spread observed between the extreme score categories.

### 3.6. Multivariable Analysis of Predictors of Lest Ventricular Hypertrophy

We performed a multivariable linear regression analysis to identify independent predictors of change in left ventricular mass (ΔLVM) over the 3-year follow-up. The model included lifestyle score (range 0–3), age, sex, blood pressure (BP) control status (controlled vs. uncontrolled), and baseline LVM as predictors. The model accounted for 32% of the variance in ΔLVM (R^2^ = 0.32). The results are summarized in Table 4.

The analysis revealed that a higher lifestyle score was associated with a reduction in ΔLVM (β = −3.25 g per unit increase, 95% CI: −5.80 to −0.70, *p* = 0.01), while uncontrolled BP was associated with an increase in ΔLVM (β = 4.80 g, 95% CI: 1.20 to 8.40, *p* = 0.01). Additionally, higher baseline LVM predicted a decrease in ΔLVM (β = −0.85 g per 10 g, 95% CI: −1.20 to −0.50, *p* < 0.001), possibly due to regression to the mean. These results suggest that lifestyle improvements and effective BP management play key roles in reducing left ventricular mass in hypertensive patients.

An additional VHD-adjusted model was undertaken to account for the biologically plausible influence of left-sided valvular lesions on left-ventricular loading (pressure/volume) and thus on LV mass trajectories, thereby strengthening internal validity. Moderate or worse left-sided VHD (mitral regurgitation/stenosis; aortic regurgitation/stenosis) at baseline was present in 16.03% of our patients (n = 21) and was added as a binary covariate alongside baseline LVM, age, sex, coronary artery disease, hypertriglyceridemia, and hyperuricemia (ordinary least squares with HC3 robust standard errors). VHD ≥ moderate was not independently associated with ΔLVM (β −7.9 g; 95% CI −45.4 to 29.5; *p* = 0.68; model R^2^ 0.12; adjusted R^2^ 0.00). Age showed a positive, non-significant trend (β +0.83 g per year; 95% CI −0.13 to 1.79; *p* = 0.09), while baseline LVM, male sex, coronary disease, hypertriglyceridemia, and hyperuricemia were not significant (all *p* > 0.18). Findings were unchanged when participants with ≥moderate VHD were excluded, and no problematic collinearity was observed.

## 4. Discussion

### 4.1. Noteworthy Results

This observational cohort study aimed to evaluate the longitudinal changes in echocardiographic parameters among hypertensive patients over a 3-year period, while also examining the influence of blood pressure control, antihypertensive medication regimens, and sustained lifestyle modifications on these changes. Our findings revealed a statistically significant increase in left ventricular mass (LVM) and left ventricular internal diameter in diastole (LVIDd) over time, accompanied by a significant rise in the prevalence of left ventricular hypertrophy (LVH), despite ongoing pharmacologic treatment. Although the absolute mean increase in LVM was modest (~9 g), it translates to an average rise of ~6 g/m^2^ in LVMI among men in our cohort. While no significant overall changes were observed in left ventricular ejection fraction (LVEF) or diastolic function (E/A ratio), patients with uncontrolled blood pressure experienced a significantly greater increase in interventricular septum (IVS) and left ventricular posterior wall (LVPW) thickness compared to those with controlled blood pressure. The class or combination of antihypertensive medications used was not significantly associated with changes in LVM or LVEF. Notably, a higher number of sustained lifestyle changes—including smoking cessation, regular exercise, and dietary weight loss—was associated with greater reductions in LVM, suggesting a potential cumulative effect of non-pharmacological interventions on cardiac remodeling. However, self-reported lifestyle changes and medication adherence are prone to subjectivity and recall bias, and our findings must be interpreted with caution.

An interesting observation in our study is the apparent discrepancy between the within-group and between-group analyses of echocardiographic changes over time. In the overall cohort, only left ventricular mass (LVM) and left ventricular internal diameter in diastole (LVIDd) showed statistically significant changes from baseline to the final follow-up, whereas interventricular septum (IVS) and left ventricular posterior wall (LVPW) thickness did not. However, when we compared the magnitude of these changes between patients with controlled and uncontrolled blood pressure, we found a different pattern: differences in IVS and LVPW changes were statistically significant, while the difference in LVM change demonstrated only a non-significant trend. This apparent contradiction is best understood by recognizing the difference in statistical approaches. The within-group analysis evaluated whether a given parameter changed significantly over time in the entire study population, regardless of blood pressure control. In contrast, the between-group comparisons assessed whether the degree of change differed between patients with controlled versus uncontrolled hypertension. As such, a variable may not change significantly across the whole cohort but still differ significantly between subgroups if one group changes more than the other. This was the case with IVS and LVPW: although their overall changes were modest and non-significant, the subgroup of patients with uncontrolled blood pressure exhibited more pronounced wall thickening, thereby producing a significant between-group difference.

An additional consideration in our study is the heterogeneity of antihypertensive treatment regimens, with most patients receiving combination therapy. While we conducted a subgroup analysis based on common medication combinations, the resulting small sample sizes in each group limit the interpretability of these findings. This complexity reflects real-world prescribing practices but also highlights the need for larger studies or stratified analyses to better isolate the effects of individual drug classes on cardiac remodeling.

### 4.2. Previous Research

Large-scale human data demonstrate that the trajectory of left-ventricular mass (LVM) depends strongly on how successfully blood pressure is lowered and which drugs are used. Early meta-analyses of strictly double-blind trials showed that agents producing the greatest systolic-BP fall—especially ACE inhibitors and, later, ARB—induced about 10−13% regression of echocardiographic LVM, whereas β-blockers and, to a lesser extent, thiazide diuretics achieved only half that effect [25,26]. A subsequent comparative meta-analysis confirmed the relative inferiority of β-blockers for LVH regression [27]. More recent longitudinal evidence echoes this pattern: in a 1872-patient Korean cohort study, every 10 mmHg drop in systolic BP translated into proportional LV-mass index reduction and LVH regression predicted fewer events [20], whereas the 18-month SPRINT-HEART substudy, despite an 11 mmHg differential, saw no significant LVM change, suggesting that structural regression may lag behind short-term intensive control [28]. Our three-year outpatient study, where just 57% of participants achieved target BP and beta-blocker/diuretic combinations predominated, instead recorded a 5% rise in mean LVM and a jump in LVH prevalence from 29% to 40%. These findings accord with the meta-analytical consensus that inadequate or pharmacologically sub-optimal BP reduction fails to reverse, and may permit progression of, hypertrophy, while the apparent discrepancy with SPRINT-HEART likely reflects its shorter follow-up and more aggressive early BP lowering. The ANBP2 Echo Study further underscores the prognostic weight of such changes, having shown that echo-defined LVH independently predicts cardiovascular outcomes in elderly hypertensive patients [29].

Structural remodeling observed in our cohort is clinically relevant because LVH elevates the risk of new-onset atrial fibrillation (AF). In a meta-analysis by Mauriello et al. (2024), myocardial oxidative stress and LV geometry independently predicted AF onset [30]. Although 15 participants in our study already had AF at baseline and were excluded from E/A analysis, no new AF cases occurred during follow-up. Nonetheless, our findings underscore the need for rhythm surveillance in SAH patients with persistent or progressive LV remodeling.

Lifestyle-directed interventions show an equally consistent, if mechanistically diverse, influence on cardiac geometry. Twelve-month sodium-restriction trials in essential hypertension achieve 5–8% LVM regression independent of drug therapy [31], and long-term weight-loss programs in overweight hypertensives yield parallel falls in ambulatory BP and LVM [32]. Exercise exerts a double-edged effect: combined aerobic training plus antihypertensive therapy lowers BP and LVM in treated hypertensives [33], whereas high-volume swim or endurance training in otherwise healthy or mildly hypertensive adults enlarges chambers and, yet, improves diastolic function [34]. Our study’s dose–response finding, that each additional sustained healthy habit trimmed approx. 3.3 g from the three-year ΔLVM, aligns with the salt-restriction and weight-loss data, reinforcing the additive value of lifestyle change beyond pharmacological BP control; the smaller absolute effect we observed is plausibly explained by more modest weight loss, partial adherence, and reliance on self-reported behaviors compared with the intensive or surgical interventions documented in the wider literature.

### 4.3. Limitations and Future Reseach Perspectives

Our study has some noteworthy limitations. As described, our sample size was too small within medication subgroups, which limited our statistical power to detect significant differences. Diastolic function assessment was limited to the E/A ratio, which is load-dependent and may not reliably reflect diastolic dysfunction without complementary parameters such as tissue Doppler imaging or left atrial volume. Echocardiograms were obtained only once a year, so intermediate remodeling trajectories and transient changes could not be captured.

Another important limitation of our study is the reliance on self-reported lifestyle modifications and medication adherence, which may be subject to recall bias. The absence of objective adherence measures limits the ability to fully attribute observed cardiac remodeling differences to sustained behavioral change.

Furthermore, the non-randomized, observational nature of our study cannot establish causation and remains vulnerable to residual confounding despite multivariable adjustment. Conducting the study in a single Romanian outpatient center with a relatively homogeneous patient pool and healthcare context limits the applicability of the results to more diverse populations.

Fourth, because speckle-tracking deformation imaging was not included in our echocardiographic protocol, parameters such as global longitudinal strain (GLS) and non-invasive myocardial-work indices were not collected; consequently, we could neither detect subtle subclinical systolic dysfunction nor assess the impact of antihypertensive therapy on myocardial mechanics, despite evidence that GLS provides incremental prognostic value in hypertensive cohorts and improves with effective treatment [35].

We also did not apply quantitative Doppler criteria for valvular heart disease beyond peak velocities, limiting conclusions about valvular contributions to LV loading.

Larger prospective studies with detailed data on medication adherence, lifestyle factors, and long-term outcomes are needed to further elucidate the complex interplay of factors influencing cardiac remodeling in hypertensive patients. This could lead to more targeted and effective interventions to prevent adverse cardiovascular events in this population.

## 5. Conclusions

In this 3-year prospective study of hypertensive outpatients, we observed a modest but statistically significant increase in left ventricular mass and LVH prevalence, despite routine clinical follow-up and pharmacologic treatment. Poor blood pressure control and lack of lifestyle modification emerged as key contributors to adverse cardiac remodeling, while patients who sustained healthy behavioral changes demonstrated smaller increases in LVM. Although our findings are consistent with the previous literature showing that inadequate or suboptimal BP control can permit progression of LVH, they also highlight the additive protective role of non-pharmacologic interventions. The observed associations reinforce the clinical value of routine echocardiographic surveillance in hypertension management and underscore the importance of targeting both hemodynamic and lifestyle determinants of cardiovascular remodeling. Given the observational design, causality cannot be inferred, and further interventional studies are needed to validate these associations. Nonetheless, our results suggest that integrated care approaches—including pharmacologic optimization and sustained lifestyle change—may help mitigate subclinical cardiac damage in hypertensive patients.

## Figures and Tables

**Table 1:** Baseline Characteristics of Study Population.

Characteristic	Value
Demographics	
Number of Patients	131
Age (years), mean ± SD	59.93 ± 15.51
Sex, n (%) Male Female	68 (51.9%) 63 (48.1%)
Provenience, n (%) Rural Urban	48 (36.6%) 83 (63.4%)
Clinical and Biological	
BMI (kg/m^2^), mean ± SD Male Female	32.15 ± 4.9 30.96 ± 3.27
Weight Category, n (%) Normal Weight Overweight Grade I Obesity Grade II Obesity Grade III Obesity	27 (20.6%) 32 (24.4%) 38 (29%) 24 (18.3%) 10 (7.7%)
Diabetes mellitus, n (%)	40 (30.5%)
Hypercholesterolemia, n (%)	63 (48.1%)
Hypertriglyceridemia, n (%)	49 (37.4%)
Coronary Artery Disease, n (%)	33 (25.2%)
Angina Grade, n (%) Grade I Grade II Grade III Grade IV	18 (13.7%) 9 (6.9%) 5 (3.8%) 2 (1.5%)
Atrial Fibrillation, n (%)	15 (11.5%)
Medication Beta-blockers, n (%) ACE inhibitors, n (%) ARBs, n (%) Diuretics, n (%) Calcium channel blockers, n (%) Statins, n (%) Antiplatelet agents, n (%) Anticoagulants, n (%) Antiarrhythmics, n (%)	85 (64.9%) 67 (51.1%) 56 (42.7%) 80 (61.1%) 48 (36.6%) 61 (46.6%) 45 (34.4%) 21 (16%) 12 (9.2%)

**Table 2:** Echocardiographical Changes Over the 3-year Follow-up Period.

Variable	Baseline Measurement Mean ± SD	Third Measurement Mean ± SD	*p*-Value
**LVM (grams)**	173.99 ± 59.33	183.26 ± 64.19	0.018
**LVMI in men (g/m** **^2^)**	103 ± 26	109 ± 28	0.037
**LVMI in women (g/m^2^)**	92 ± 24	97 ± 26	0.027
**LVEF (%)**	54.51 ± 6.57	54.56 ± 7.18	0.585
**E/A Ratio**	1.06 ± 0.41	1.02 ± 0.44	0.095
**LVIDd (cm)**	4.65 ± 0.56	4.72 ± 0.62	0.012
**IVS (cm)**	1.026 ± 0.19	1.062 ± 0.18	0.109
**LVPW (cm)**	1.042 ± 0.16	1.05 ± 0.17	0.567

**Table 3:** Change in Left Ventricular Mass by Lifestyle Score Group.

Lifestyle Score	Description	n	Median ΔLVM (g)	Interquartile Range (IQR)
0	No sustained lifestyle changes	61	7.5	[+2.1, +9.9]
1	One sustained change (e.g., exercise only)	24	1	[−2.3, +3.3]
2	Two sustained changes	27	−2.2	[−6.8, +0.9]
3	All three changes (smoking cessation, diet, exercise)	19	−4.3	[−8.7, +0.2]

**Table 4:** Predictors of Change in LVM.

Predictor	Coefficient (β)	95% CI	*p*-Value
Lifestyle score (per unit increase)	−3.25	(−5.80, −0.70)	0.01
Age (per year)	0.12	(−0.05, 0.29)	0.16
Sex (male vs. female)	1.50	(−2.10, 5.10)	0.41
BP control status (uncontrolled vs. controlled)	4.80	(1.20, 8.40)	0.01
Baseline LVM (per 10 g increase)	−0.85	(−1.20, −0.50)	<0.001

## Data Availability

The original contributions presented in the study are included in the article. Further inquiries can be directed to the corresponding author.
